# Evaluating the Impact of 2D MRI Slice Orientation and Location on Alzheimer’s Disease Diagnosis Using a Lightweight Convolutional Neural Network

**DOI:** 10.3390/jimaging11080260

**Published:** 2025-08-05

**Authors:** Nadia A. Mohsin, Mohammed H. Abdulameer

**Affiliations:** 1 Department of Computer Science, Faculty of Computer Science and Mathematics, University of Kufa, Najaf 54001, Iraq; 2 Department of Computer Science, Faculty of Education for Women, University of Kufa, Najaf 54001, Iraq; mohammed.almayali@uokufa.edu.iq

**Keywords:** Alzheimer’s disease, deep learning, lightweight, CNN, MRI

## Abstract

Accurate detection of Alzheimer’s disease (AD) is critical yet challenging for early medical intervention. Deep learning methods, especially convolutional neural networks (CNNs), have shown promising potential for improving diagnostic accuracy using magnetic resonance imaging (MRI). This study aims to identify the most informative combination of MRI slice orientation and anatomical location for AD classification. We propose an automated framework that first selects the most relevant slices using a feature entropy-based method applied to activation maps from a pretrained CNN model. For classification, we employ a lightweight CNN architecture based on depthwise separable convolutions to efficiently analyze the selected 2D MRI slices extracted from preprocessed 3D brain scans. To further interpret model behavior, an attention mechanism is integrated to analyze which feature level contributes the most to the classification process. The model is evaluated on three binary tasks: AD vs. mild cognitive impairment (MCI), AD vs. cognitively normal (CN), and MCI vs. CN. The experimental results show the highest accuracy (97.4%) in distinguishing AD from CN when utilizing the selected slices from the ninth axial segment, followed by the tenth segment of coronal and sagittal orientations. These findings demonstrate the significance of slice location and orientation in MRI-based AD diagnosis and highlight the potential of lightweight CNNs for clinical use.

## 1. Introduction

Alzheimer’s disease is an age-related disease that causes damage to the brain nerve cells (neurons) responsible for primary human activities such as remembering, speaking, and thinking. Once symptoms appear and the clinical diagnosis is confirmed, the disease becomes irreversible, making a diagnosis at its early stages an urgent requirement for medical intervention to slow down its progression [1]. AD progresses in three stages: preclinical AD with brain biomarkers, MCI, and dementia due to AD [2,3]. Although MCI is commonly associated with AD, not all patients develop the disease. While AD can be confirmed after death by histopathological examination, it can also be assessed during an individual’s lifetime using clinical examination and neuroimaging techniques [1,2,3].

Magnetic resonance imaging is the most commonly used neuroimaging technique in AD research, due to its high-resolution visualization that provides related details such as hippocampal atrophy and cortical thinning [1,4,5]. Additionally, MRI is non-invasive and safe even if repeated many times, making it ideal for long-term disease progression monitoring [6,7]. However, processing 3D MRI images is computationally extensive, as each image contains millions of voxels, leading researchers to explore efficient methods to minimize the computational overhead, as in [3,8].

Deep learning techniques, particularly CNNs, have been intensely investigated in AD research as they can automatically extract relevant features rather than depending on handcrafted ones. The integration of CNNs with 3D and 2D MRI has shown relatively good results in distinguishing AD from MCI and CN states [9,10,11,12,13].

MRI scans are typically visualized using three standard volumetric orientations: axial, coronal, and sagittal. Each orientation offers a different perspective of the brain’s structure. At the same time, a particular orientation of a specific brain location may provide more critical information in distinguishing and identifying AD-related changes. Investigating the influence of these factors is crucial for maximizing neuroimaging-based models. Although MRI slice orientation and selection significantly impact classification performance, they have not been extensively studied and there are many gaps in existing studies. The literature in this area relies on investigating a single orientation or specific brain segment or using complicated machine learning techniques for the feature extraction phase. Thus, we can summarize the limitations as follows:

- Limited comparisons across various orientations of the brain;
- Lack of clear definitions for guided selection of brain slices;
- The literature suffers from complicated models.

Therefore, in this study, we present a lightweight deep learning model to investigate the impact of brain slice orientation and anatomical segmentation on AD classification. The model employs depthwise separable convolutions, significantly reducing training time without sacrificing performance. To enhance the diagnostic relevance of the input data, we introduce an automated slice selection mechanism based on feature entropy computed from a pretrained CNN (MobileNetV2) activation map. This method systematically selects the most informative slice from each 3D MRI segment across axial, coronal, and sagittal views. The key contributions of this research are summarized as follows:

- A comprehensive analysis of the influence of MRI slice orientation on AD classification;
- Introduction of a feature entropy-based slice selection method that identifies the most informative slice per brain segment using CNN activation maps;
- Development of a simple yet effective lightweight CNN model based on depthwise separable convolutions for efficient classification;
- Brain slice analysis using a consistent partitioning strategy to evaluate region-wise diagnostic importance;
- Introducing an attention mechanism for feature level interpretation.

This study provides a foundation for further investigation into MRI-based AD diagnosis by identifying optimal combinations of slice orientation and location for enhanced classification accuracy.

## 2. Related Work

Many studies have investigated AD detection using deep learning techniques, focusing on neuroimaging, in which MRI and positron emission tomography (PET) scans have received the highest interest. In this study, we focused on MRI scans. It is essential to effectively use the available 3D MRIs for Alzheimer’s disease diagnosis in a way that facilitates future research through identifying the most relevant 2D slices and orientations to be extracted from these 3D scans. Most previous studies in this domain have relied heavily on random slice selection or focused on a specific brain orientation, while others have employed complex and extensive machine learning techniques to specify relevant slices. We also noticed that some achieved relatively low accuracy when compared to other state-of-the-art methods.

Kim et al. [14] proposed a notable effort: a relevant slice-selective method that relies on a generative adversarial network for PET scans. The brain area considered in their research ranges from the amygdala to the end of the posterior cingulate cortex (PCC). They chose this specific brain part as it is the part where AD causes anatomical and pathological changes. Their model is a binary classifier that classifies AD vs. CN, which was trained using the selected slices on the coronal plane without considering axial and sagittal orientations. They achieved 92% and 94% accuracy using single and double slices, respectively.

Similarly, focusing on slice orientation, Ramalho et al. [4] highlighted the importance of MRI orientations over the AD diagnosis by studying the impact of different orientations on classifying patients with MCI versus healthy CN patients. The evaluation was conducted using a CNN model. While all three orientations were investigated, the study did not specify the particular slices or brain regions analyzed within each orientation. The classification only focused on MCI vs. CN without considering the AD stage.

From a different perspective, Puente-Castro et al. [15] focused on studying the sagittal plane, which is less commonly utilized compared to other horizontal orientations. They employed transfer learning to assess the sagittal plane, and their study showed that it is equally effective compared to the other horizontal planes. However, the authors did not specify which brain region was employed in their research.

In contrast, De Souza et al. [16] introduced a hybrid model that combined machine and transfer learning for AD prediction from MRI scans. They employed a genetic algorithm for slice selection. Once the relevant slices were selected, transfer learning using EfficientNetV2S was utilized for feature extraction and classification. The experiments were applied to male and female MRIs separately and combined. AD, CN, and MCI were the three classes considered. First, a multiclass classification was used, in which AD vs. all, MCI vs. all, and CN vs. all were considered. Three brain planes were considered, and the slice selection pipeline specified the relevant slices from all the orientations and chose adjacent slices only.

A similar hybrid model consisting of machine learning and a deep learning pipeline for slice selection from 3D MRI images was proposed by Inan et al. [17]. Random forest and gradient boosting were utilized to select the 16 most relevant slices. A transfer learning model based on EfficientNetV2S extracted features and classified subjects using binary classification of CN vs. AD, CN vs. MCI, and CN vs. MCI, achieving an accuracy of 83.64%, 82.69%, and 71.40%, respectively.

Ghosh et al. [18] proposed a deep learning model to be trained in a federated way to detect Alzheimer’s disease using MRI images. Privacy, consistency, and robustness were tackled in this research. The initial weight of MobileNet, which was gained from training the ImageNet database, was used. MRI slice orientations, coronal, sagittal, and axial planes, were explored to choose the best orientation for AD detection. However, the authors did not specify which coronal slice was selected for model training, leaving room for further exploration regarding the most informative regions within the coronal plane.

Beyond MRI scans, Khatri et al. [19] integrated MRI and functional MRI (fMRI) to identify AD biomarkers. A support vector machine (SVM) and a random forest (RF) were used to classify the data. They worked with a specific frequency range and considered 48 axial slices of the fMRI. Finally, Bi et al. [6] employed unsupervised technologies. First, they employed PCANet, an unsupervised CNN, which was used for feature extraction, and then k-means for AD diagnosis. Table 1 summarizes the most relevant articles investigating the influence of different orientations and slice selection methodologies for AD classification. It compares the used datasets, methods, orientations, and datatypes.

## 3. Materials and Methods

**Figure 1:**
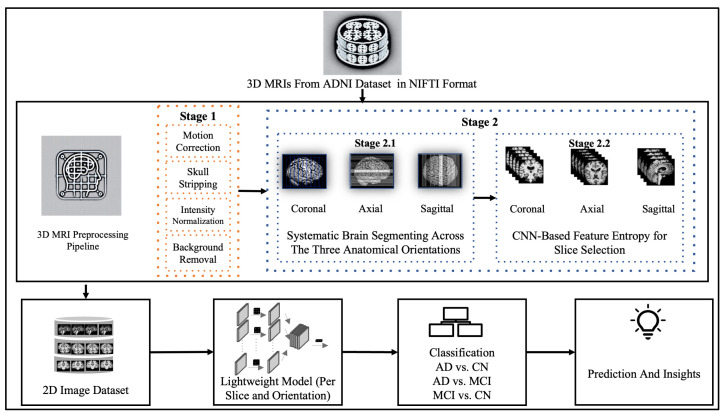
Overview of the research workflow.

This section describes the dataset and methodologies used. Figure 1 illustrates the overall framework of this study, beginning with data acquisition and preprocessing, followed by the selection of 2D slices for the three primary brain orientations. Next, selected slices from each partition and orientation were fed into the lightweight CNN to gain insights about the best combination for Alzheimer’s disease detection.

### 3.1. The Alzheimer’s Disease Neuroimaging Initiative (ADNI) Dataset

The ADNI is a large research center project mainly launched to study biomarkers leading to early AD detection. ADNI significantly contributed to the studies in this area using machine and deep learning by offering large and high-resolution neuroimaging. It is recognized internationally as a trusted source of experimental data on Alzheimer’s disease. Permission is needed to access the ADNI dataset. In this study, we applied to access the data through the Image and Data Archive (IDA) and received our approval on 21 June 2023. From the ADNI1 collection, we downloaded 3495 3D MRI scans. The scans were divided into AD, MCI, and CN, as shown in Table 2.

### 3.2. MRI Preprocessing

Preparing medical images for analysis is a critical step that facilitates the development of effective deep learning models. Many preprocessing procedures must be implemented to prepare medical images for the proposed architecture. To ensure accurate classification results, we prepared the 3D MRI images obtained from the ADNI dataset for further processing and analysis. We utilized FreeSurfer, a well-known open-source tool mainly designed to process structural MRI scans. The Autorecon1 pipeline was applied to perform essential procedures, including (1) motion correction, (2) skull stripping, and (3) intensity normalization, and we added one more non-FreeSurfer process: (4) background removal. These four processes are explained as follows:

1. Motion correction corrects minor head movements that may occur during head scanning. It ensures that all slices in one scan are aligned.
2. Skull stripping removes all non-brain tissues from MRI scans to isolate the brain, which is a crucial step for AD classification.
3. Intensity normalization adjusts the intensities of MRI voxels to a standard range to enhance the contrast and consistency in the images.
4. For background removal, we cropped the background to eliminate most of the non-brain elements.

**Figure 2:**
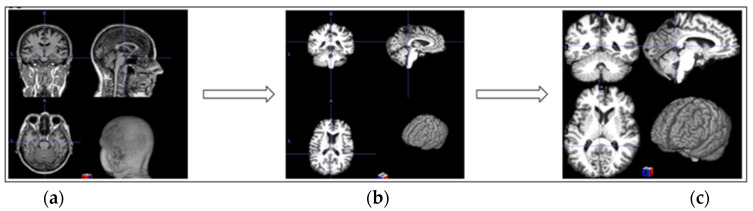
**Three-dimensional** MRI preprocessing: (**a**) original image; (**b**) preprocessed with FreeSurfer autorecon1; (**c**) background removal.

Although FreeSurfer is widely used for processing medical images, it is considered time-consuming. Our device’s processing time for a single image with autorecon1 took approximately 30 min. Given that our dataset contained over 3450 images, we would have needed more than 1700 h, nearly 70 days, to preprocess all the images. To address this problem, we implemented parallel processing using eight cores, which significantly reduced the preprocessing time, thereby making the pipeline more efficient for large-scale analysis. Figure 2 shows the preprocessing stage.

### 3.3. Automated 2D Slice Selection

To enable consistent analysis of the influence of 2D MRI slice orientation and location across subjects in AD diagnosis, we developed an automated slice selection framework based on entropy computed from CNN feature maps. This method identifies the most informative slices from 3D MRI volumes while preserving anatomically and diagnostically relevant structures.

For each 3D image, $I\in R^{X\times Y\times Z}$ was partitioned into fifteen equally sized segments along the three standard orientations: Axial, Coronal, and Sagittal. Let V denote a normalized 2D MRI slice extracted from a segment S, and let FE denote Feature Extractor from the CNN. The 2D slices V ∈ $R^{W\times H\times 3}$ are passed through the CNN to obtain a 3D activation map as in (1):

(1) $$A=FE\left(V\right)\in R^{H^{\prime }\times W^{\prime }\times C}$$

where C is the number of feature channels. For each channel A_c_, we compute the Shannon entropy as shown in (2):

(2) $$H\left(A_{c}\right)=-\sum_{i}P_{i}\log P_{i}$$

where $P_{i}$ is the estimated probability from the normalized histogram of pixel intensities in $A_{c}$. Equation (3) explains the overall entropy of slice V, which is defined as the average entropy across all channels:

(3) $$H_{mean}\left(V\right)=\frac{1}{C}\sum_{c=1}^{C}H\left(A_{c}\right)$$

The slice with the highest mean entropy is selected as the most informative representative of that segment:

(4) $$V^{*}=\max\left(H_{mean}(v)\right)$$

In the sagittal plane, the brain was divided from left to right into vertical slices along the midline. In the axial plane, the brain was segmented from top to bottom, resulting in horizontal cross-sections. For the coronal plane, the division proceeded from the back to the front of the brain, resulting in vertical slices that moved from the back of the skull toward the forehead. This segmentation approach allows the model to identify the most informative slice from each region independently by narrowing the search space within defined anatomical brain segments. This method reduces the risk of overlooking critical features that may be missed in global analysis. Moreover, it enhances the interpretability and relevance of the selected slices.

**Figure 3:**
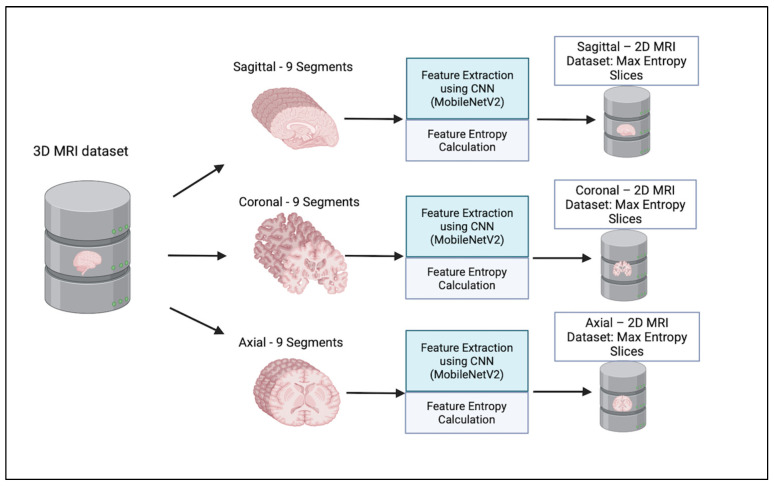
**Two-dimensional** MRI Slice selection process.

**Figure 4:**
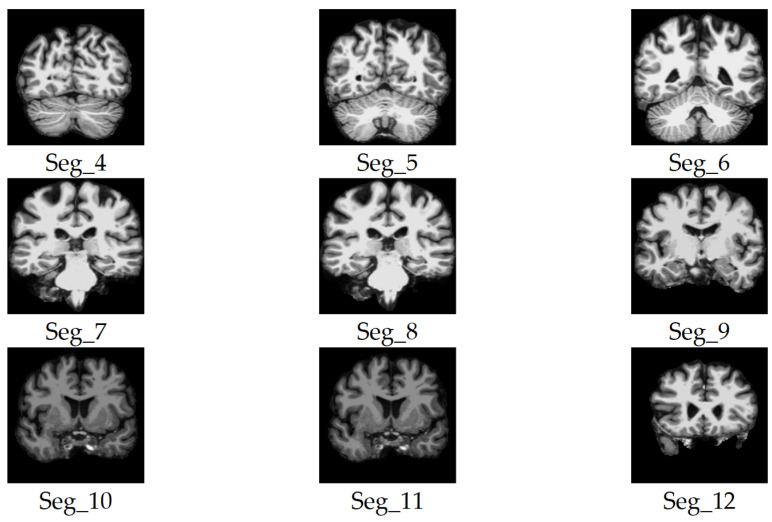
Samples of the selected slices of the coronal plane segments.

**Figure 5:**
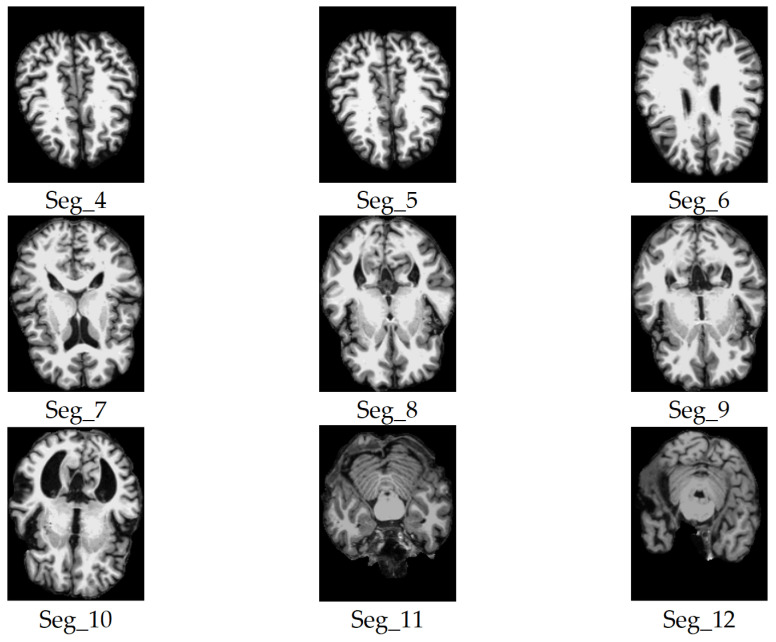
Samples of the selected slices of the axial plane segments.

**Figure 6:**
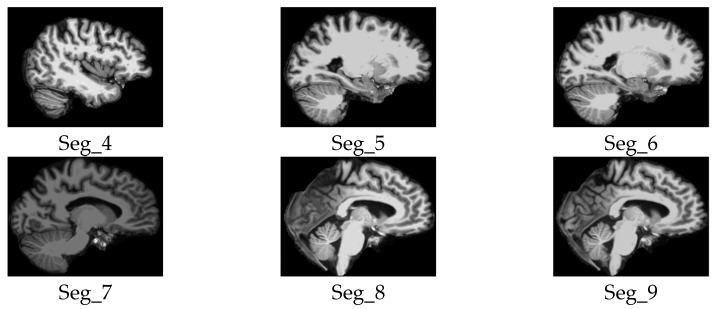

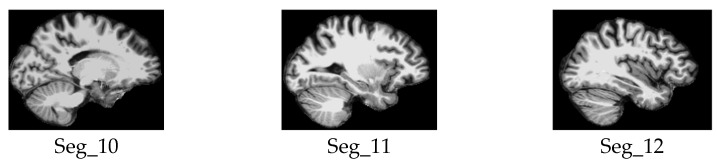
Samples of the selected slices of the sagittal plane segments.

We considered only the nine central segments, neglecting the edge parts of the brain, as they did not contain complete brain images, but rather partial structures, which limits their diagnostic value. Figure 3 illustrates the 2D slice selection process. Figure 4, Figure 5 and Figure 6 show samples of the selected 2D images.

### 3.4. Proposed Lightweight CNN with Attention-Based Fusion

Our primary goal was to create a lightweight and efficient model designed to classify patients into three main categories: patients with AD vs. CN, AD vs. MCI, and, finally, MCI vs. CN. Focusing on these critical comparisons aimed to answer our research question and identify the most impactful orientation and brain parts contributing to precise diagnostic outcomes. Additionally, by leveraging attention-based feature fusion, the model offers interpretability, highlighting which level of features influenced the decision, thereby enhancing clinical relevance.

The proposed architecture is a multi-layered network built on a depth-wise separable CNN, which serves as the model backbone. The choice of a lightweight design was motivated not only by the need to reduce computational overhead but also by a commitment to the principles of sustainable artificial intelligence (Green AI). By significantly reducing the number of floating-point operations (FLOPs), the model minimizes energy consumption, which in turn helps lower carbon emissions.

**Figure 7:**
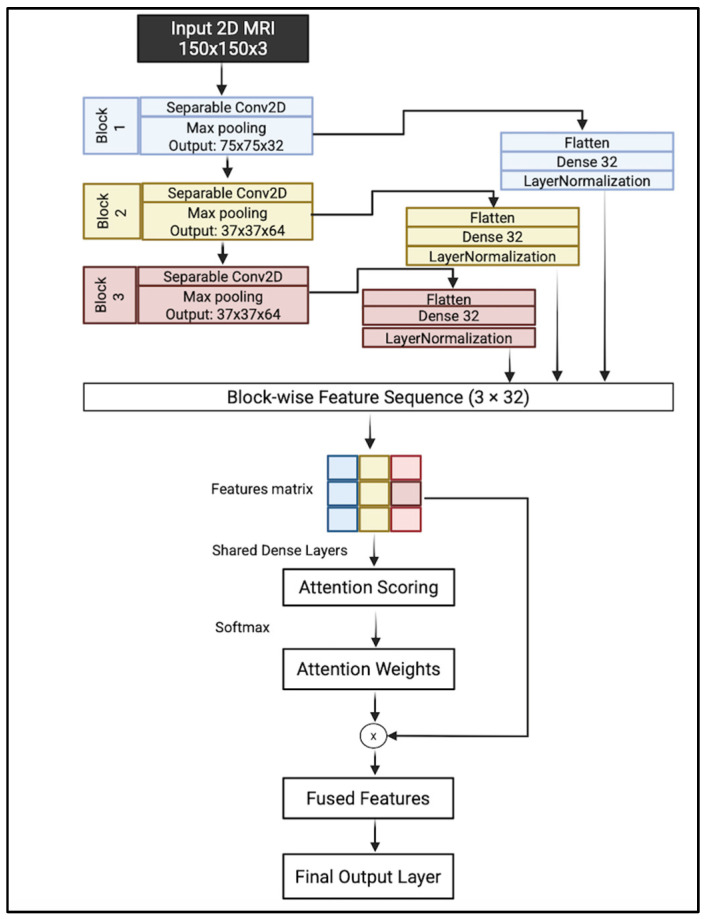
Complete overview of the proposed model.

We proposed a lightweight convolutional neural network architecture enhanced with hierarchical attention fusion to improve Alzheimer’s Disease classification from 2D MRI slices. The model utilizes depthwise separable convolutions and a custom attention mechanism to reduce computational cost while maintaining spatial feature relevance. A complete overview of the architecture is illustrated in Figure 7.

A hierarchical feature extraction approach is introduced instead of relying on the last layer. The model collects and integrates features from multiple levels of abstraction, which helps in capturing both fine-grained structural details and broader anatomical patterns.

The proposed architecture takes an input image of size 150 × 150 × 3. It consisted of three sequential convolutional blocks; each block consisted of depthwise separable filters with 3 × 3 kernels followed by a max-pooling layer with a kernel size of 2 × 2. Each block captures a distinct level of abstraction:

- Block 1 focuses on low-level visual features such as edges, textures, and intensity gradients;
- Block 2 captures mid-level representations, including shapes and localized anatomical patterns;
- Block 3 encodes high-level semantic information, such as complex structures or regions.

This multiscale feature extraction enables the model to analyze brain MRI slices by learning attention weights across these blocks and emphasizing the most relevant level of abstraction for each input. The output of each block is flattened and projected into a shared 32-dimensional latent space using fully connected layers. These feature vectors are normalized to ensure consistent statistical properties across blocks.

To determine the relative importance of each block’s features, an attention mechanism is introduced. The normalized vectors are stacked along a new temporal axis, forming a feature matrix. This matrix is processed through shared dense layers, producing attention logits for each block. These layers learn higher-level abstract representations of each block’s features, preparing them for attention scoring. A Softmax is applied across the three blocks to produce attention weights that sum to one, enabling the network to learn a weighted fusion of features based on relevance to the classification task. These weights are then used to perform element-wise multiplication with the corresponding feature vectors, allowing the model to emphasize the most informative feature level.

The attention-weighted vectors are aggregated via summation to produce a fused feature representation, which is then passed through a final dense layer with sigmoid activation to generate the binary classification output.

#### Depthwise Separable Convolution (DSC)

**Figure 8:**
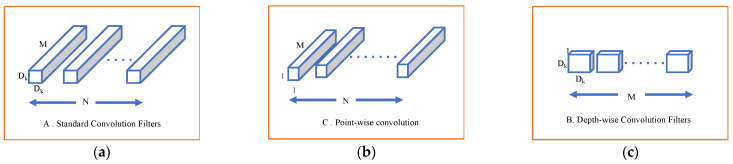
(**a**) Standard CNN filters; (**b**) depth-wise convolution; (**c**) point-wise convolution.

Our model employs depthwise separable convolution. This type of deep learning convolution is specially designed to reduce the computational complexity and number of parameters in conventional CNN. DSC factorizes the standard CNN into point- and depth-wise convolutions. A depthwise convolution mainly uses a single convolutional filter per input instead of combining all input channels as in standard convolution. In contrast, the point-wise convolution, which is a 1 × 1 convolution, combines the output of the previous layer. Figure 8 illustrates the depth- and point-wise convolution. By applying this separation, DCS dramatically reduces the computation cost, as shown in (5) for standard CNN and (6) for DSC [20].

(5) $$D_{k}\times D_{k}\times M\times N\times D_{f}\times D_{f}$$

(6) Depth-wiseconvolution Point-wiseconvolutionDk×Dk×M×Df×Df⏞+M×N×Df×Df⏞

In these expressions, *D_k_* represents the kernel size, *M* is the number of input channels, *N* is the number of output channels, and *D_f_* is the spatial dimensions (height × width) of the feature map.

## 4. Experimental Results

### 4.1. Experimental Environment and Platform

All experiments were conducted using Python 3.11.7 within Jupyter Notebook(via Anaconda distribution, notebook server version 6.5.7) on a MacBook Pro with an Apple M1 chip, 10 cores, 16 GB of memory, and macOS Sonoma. The model was trained using the Adam optimizer with a learning rate of 0.001. Binary cross-entropy was used as the loss function. Training was conducted over 20 epochs with a batch size of 32. A 5-fold stratified K-Fold cross-validation strategy was applied, with 10% of the data held out for testing in each fold.

### 4.2. Evaluation Metrics

Before discussing the performance metrics used, it is essential to understand four fundamental terms: true positive (TP), true negative (TN), false positive (FP), and false negative (FN). These terms define the accuracy of the predictions made by our model, where MRI scans from the ADNI dataset were classified into three main classes: AD, MCI, and CN.

- AD vs. MCI
  ○ TP: The patient is correctly predicted as having AD when they have Alzheimer’s disease.
  ○ TN: The patient is correctly predicted as having MCI when they have a mild cognitive impairment.
  ○ FP: The model incorrectly predicts AD when the patient has mild cognitive impairment.
  ○ FN: The model incorrectly predicts MCI when the patient has Alzheimer’s disease.
- AD vs. CN
  ○ TP: The patient is correctly predicted as having AD while they have Alzheimer’s disease.
  ○ TN: The patient is correctly predicted as CN when they are cognitively normal.
  ○ FP: The model incorrectly predicts AD when the patient is cognitively normal.
  ○ FN: The model incorrectly predicts CN when the patient has Alzheimer’s disease.
- MCI vs. CN
  ○ TP: The patient is correctly predicted as having MCI when they have mild cognitive impairment.
  ○ TN: The patient is correctly predicted as CN when they are cognitively normal.
  ○ FP: The model incorrectly predicts MCI when the patient is cognitively normal.
  ○ FN: The model incorrectly predicts CN when the patient has mild cognitive impairment.

Several performance metrics were utilized to draw attention to the importance of different orientations and slice locations for AD diagnosis. First, the computational load was assessed by calculating the number of FLOPs. In addition, an attention-based feature importance analysis was conducted to understand which blocks the model relies on most in the classification process. Subsequently, each classification experiment was evaluated using standard performance metrics, including accuracy, precision, sensitivity (recall), specificity, F1 score, and the area under the receiver operating characteristic curve (AUC).

1. Accuracy represents the number of correctly classified MRIs (*TP* and *TN*) out of all the predictions shown in Equation (7).

(7) $$Accuracy=\frac{TP+TN}{TP+TN+FP+FN}$$

2. Precision represents the number of positively predicted MRIs out of all positive predictions (*TP* and *FP*), as shown in Equation (8).

(8) $$Precision=\frac{TP}{TP+FP}$$

3. Sensitivity measures the model’s ability to detect positive cases (patients with AD and MCI). High sensitivity is a significant measure in the medical field [18] since it means how well the model identifies actual disease cases, reducing the *FN* rate, as shown in Equation (9).

(9) $$Sensitivity=\frac{TP}{TP+FN}$$

4. Specificity measures the model’s ability to detect true negative (non-AD) cases. It is essential to minimize the *FP* in clinical diagnosis, and it is calculated as in Equation (10).

(10) $$Specificity=\frac{TN}{TN+FP}$$

5. The *F*1 score is a harmonic balance between precision and sensitivity. The main reason for considering the *F*1 score in the performance evaluation process is that we are working in a sensitive medical area [21], which can be calculated using Equation (11).

(11) $$F1=2\times \frac{precision\times sensitivity}{precision+sensitivity}$$

6. The AUC assesses the model by plotting the TP rate (TPR) to the FP rate (FPR).

### 4.3. Experimental Results Results Discussion

To benchmark our approach, we compared our model with three known lightweight architectures—MobileNetV1, MobileNetV2, and EfficientNetB0, based on their computational cost, as measured by the number of FLOPs as shown in Table 3. The proposed method stands out with the lowest FLOPs, requiring approximately 78.1 million operations. It reflects a significant reduction in computational complexity—about 74% less than MobileNetV2 and over 80% less than MobileNetV1 and EfficientNetB0.

**Figure 9:**
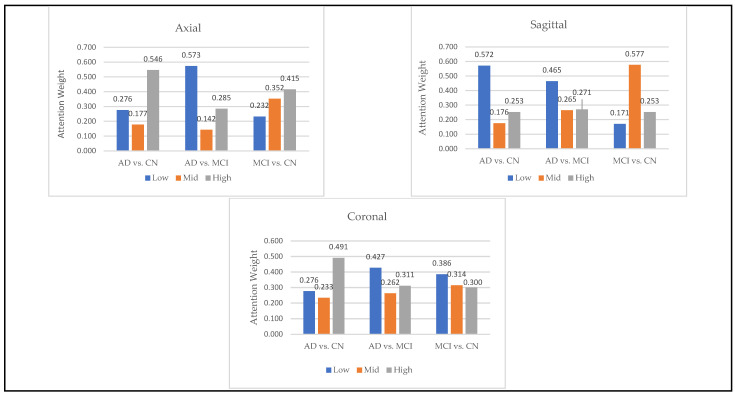
Contribution of Low, Mid, and High Feature Levels in Alzheimer’s Classification Tasks.

To analyze the role of hierarchical features, we calculated the average of attention weights assigned to low, medium, and high-level features across segments as shown in Figure 9. These correspond to features extracted from Block 1 (low-level), Block 2 (mid-level), and Block 3 (high-level) in the convolutional architecture. The results revealed that high-level features were most impactful for AD vs. CN classification, especially in axial and coronal orientations, whereas mid-level features played a larger role in differentiating MCI from CN in sagittal views. In the AD vs. MCI tasks, the model relied more on low-level features, suggesting that fine spatial details—like edges and textures—may be helpful in capturing the subtle differences between these two conditions.

**Figure 10:**
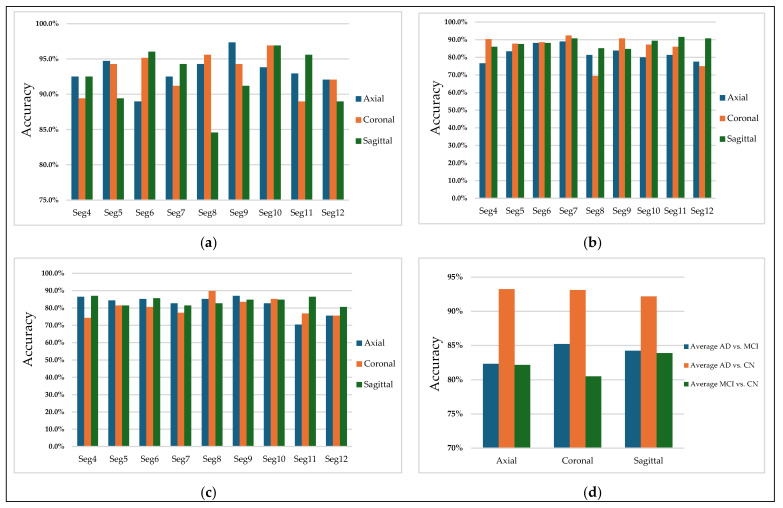
Classification accuracy for (**a**) AD vs. CN, (**b**) AD vs. MCI, (**c**) MCI vs. CN, and (**d**) Average accuracy across all slices.

Figure 10 highlights the classification accuracy for different slices and illustrates the effect of orientations on the model’s performance. The first three subplots (a), (b), and (c) are for the binary classification tasks: AD vs. MCI, AD vs. CN, and MCI vs. CN, respectively. Each subplot shows the accuracy plotted against multiple 2D MRI selected slices of nine segments (Seg-4 to Seg-12) for the sagittal, coronal, and axial orientations. In contrast, the fourth subplot (d) represents the average of the three orientations across all slices.

**Figure 11:**
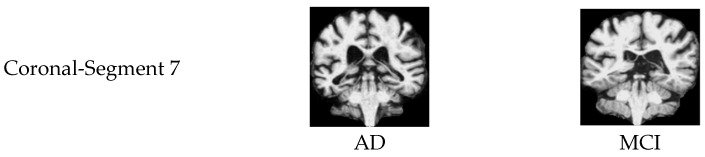
Samples of 2D MRI slices achieving the highest classification accuracies for AD vs. MCI.

Subplot (a), corresponding to AD vs. MCI, shows that the selected slices from segments 7 of the coronal plane have more discriminative features in distinguishing Alzheimer’s disease and mild cognitive impairment patients, achieving an accuracy of 92%. Figure 11 illustrates sample images that achieved higher accuracies in classifying AD vs. MCI.

**Figure 12:**
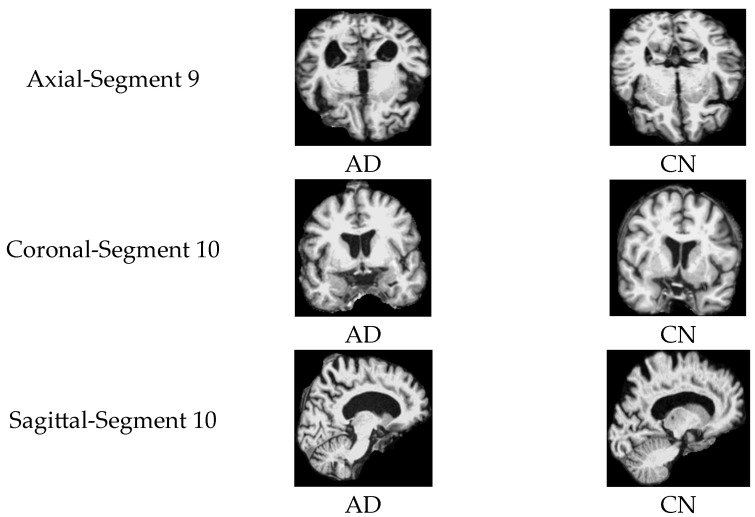
Samples of 2D MRI slices achieving the highest classification accuracies for AD vs. CN.

Subplot (b), which corresponds to the AD vs. CN classification task, indicates that the highest accuracy values, reaching 97%, were achieved with three specific orientation–slice combinations: axial plane with segment 9, coronal and sagittal plane with segment 10. Figure 12 shows some samples of those images. The above results suggest that these orientation–segment combinations have more discriminative features for distinguishing Alzheimer’s disease from cognitively normal subjects than the others, as evidenced by the higher classification accuracy achieved in these cases. The graph further illustrates that the center segments of the brain play the most significant role in the classification process on the axial and coronal planes. The opposite happens on the sagittal plane, where the edge segments have a higher effect on the prediction process.

Notably, the best-performing segments identified by our model, such as the ninth axial segment, yielded the highest classification accuracy in distinguishing AD from CN. The strong performance of the ninth axial segment in distinguishing AD from CN can be attributed to its anatomical coverage of the medial temporal lobe, including the hippocampus and surrounding structures regions commonly affected in early Alzheimer’s pathology. It is further supported by the attention weights, which show that high-level features contributed most significantly to classification (49.1%), suggesting that the model relied on disease-relevant atrophic patterns present in this slice.

**Figure 13:**
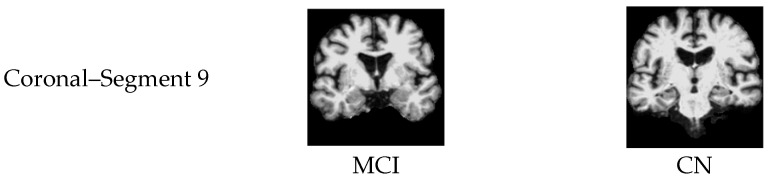
Samples of 2D MRI slices achieving the highest classification accuracies for MCI vs. CN.

In general, MCI vs. CN, shown in subplot (c), yielded the lowest accuracy values compared to the other classification processes. The Coronal plane played the most significant role in identifying MCI patients. Figure 13 illustrates samples of 2D MRI slices that achieved higher accuracies in classifying MCI vs. CN.

Finally, subplot (d) illustrates a comparative analysis of the average accuracy of all slices for the three orientations. AD vs. CN shows the highest average accuracy among all selected slices from all segments, suggesting that distinguishing Alzheimer’s disease from cognitively normal individuals is more straightforward using CNN-based features.

**Figure 14:**
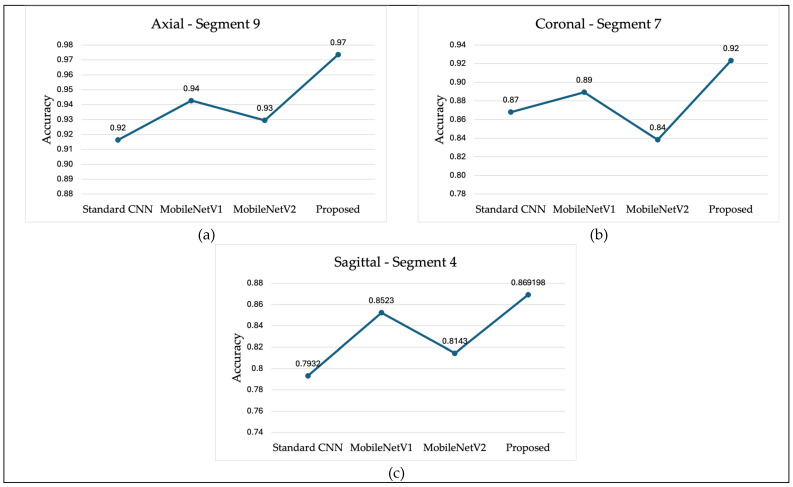
Classification Accuracy Comparison (**a**) AD vs. CN, (**b**) AD vs. MCI, (**c**) MCI vs. CN.

Figure 14 presents a comparison of classification accuracy between the proposed method and several state-of-the-art models, including MobileNetV1, MobileNetV2, and a conventional three-layer CNN. Subfigure (a) illustrates the classification accuracy for the AD vs. CN task using axial plane slices from Segment 9. Subfigure (b) shows the performance for the AD vs. MCI task using coronal plane slices from Segment 7. Lastly, subfigure (c) compares the results for the MCI vs. CN task using sagittal plane slices from Segment 4. In all three comparisons, the proposed model achieved higher classification accuracy than the standard CNN and both versions of MobileNet. Notably, MobileNetV1 generally performed better than MobileNetV2 and the standard CNN.

Table 4, Table 5 and Table 6 present detailed results for the accuracy, precision, sensitivity, specificity, F1 score, and AUC for the comparisons of AD vs. CN, AD vs. MCI, and MCI vs. CN, respectively. Table 4 illustrates that, as previously mentioned, Segment 9 in the axial orientation obtained the highest accuracy. Axial and coronal slices showed comparable average accuracies (93.2 and 93.1, respectively), slightly outperforming the sagittal view (92.2).

Table 5 shows that among the three orientations, coronal slices achieved the highest average accuracy (85.2), with Segment 7 performing best, followed closely by Segment 9. The sagittal plane also showed strong performance in Segments 10 and 11. In contrast, axial slices demonstrated comparatively lower average accuracy, with Segment 7 being the most discriminative.

Table 6 shows that, on average, sagittal slices achieved the highest accuracy, specifically for segments 4, 6, and 11. The axial orientation also yielded comparable results, with an average accuracy of 82.2, particularly in Segment 9. Meanwhile, coronal slices had the lowest average accuracy, although Segment 8 achieved an accuracy of 89.9. These results suggest that mid-to-edge segments in the sagittal and axial planes are more informative for distinguishing MCI from CN.

## 5. Conclusions

In this study, we investigated the impact of 2D MRI slice orientations, along with the segment location of the selected slice, on the performance of a lightweight CNN model for AD diagnosis. The used images were derived from 3D MRIs obtained from the ADNI dataset, across the three MRI orientations: axial, coronal, and sagittal. The model’s performance was evaluated using different performance metrics. The results demonstrated a clear influence of both the orientation and the slice position. The model performed the best in distinguishing AD from CN subjects; in particular, among all configurations, the axial slices yielded the most consistent performance across multiple segments (particularly segment 9), where it achieved the highest overall accuracy of 97.4%. The coronal slices also demonstrated strong results in segments 8 through 11. In contrast, the sagittal orientation performed best in segment 10, with an accuracy of 96.9%.

In conclusion, this work demonstrates that the combination of slice orientation and slice location has a significant impact on classification performance, and that a careful choice of these parameters can enhance model performance for lightweight CNN-based Alzheimer’s disease classification. Future research may extend these findings by incorporating the most significant segments to explore cross-orientation fusion strategies or validating the results on larger multi-site datasets to assess their generalizability.

## Figures and Tables

**Table 1:** Summary of the related work.

Article	Used Dataset	Data Type	Methods	Classification	Selected Slices	Orientations
Kim et al. [14]	ADNI and Severance Hospital	CT + PET	GAN + SVM	AD vs. CN	Double slices covering the PCC	Coronal
Ramalho et al. [4]	ADNI	MRI	CNN	MCI vs. CN	Not explicitly specified	All
Puente et al. [15]	ADNI + OASIS	MRI + AGE and SEX	ResNet + SVM	Multi-class (AD, CN, and MCI)	Not explicitly specified	Sagittal
de Souza et al. [16]	ADNI	MRI	Genetic algorithms + EfficientNet	CN vs. ALL, AD vs. ALL, and MCI vs. ALL	From 67 to 71 and 65 to 69 with BET on sagittal From 58 to 61 on sagittal, 10 on coronal and without BET	All
Inan et al. [17]	ADNI + OASIS	MRI	K-means, gradient boosting, random forest, and EffecientNet	CN vs. AD, CN vs. MCIc, and CN vs. MCInc	Not explicitly specified	All
Ghosh et al. [18]	ADNI + OASIS	MRI + AGE and SEX	DensNet, InceptionResNet, ResNet, and MobileNet	AD vs. AD	Not explicitly specified	All
Khatri et al. [19]	ADNI	MRI + rs-fMRI	SVN, random forest, PCANet, and k-means	AD vs. CN, CN vs. MCI, AD vs. MCI, and MCIs vs. MCIc	Forty-eight axial slices	Axial

**Table 2:** Details of ADNI subjects.

Diagnosis Stage	Total Number of 3D MRI Images	Training 80%	Validation 10%	Testing 10%
AD	1125	900	112	113
CN	1146	916	115	115
MCI	1224	979	122	123

**Table 3:** FLOPs Comparison of Proposed and Baseline Lightweight Models.

Method	FLOPs
MobileNetV1	467,840,993
MobileNetV2	301,415,457
EfficientNetB0	397,007,176
Proposed Model	78,102,649

**Table 4:** AD vs. CN.

Orientation	Segment	Accuracy	Precision	Sensitivity	Specificity	F1 Score	AUC
Axial	Segment 4	92.5	94.4	90.2	92.2	94.8	97.0
	Segment 5	94.7	92.4	97.3	94.8	92.2	98.7
	Segment 6	89.0	85.4	93.8	89.4	84.3	94.7
	Segment 7	92.5	91.3	93.8	92.5	91.3	94.8
	Segment 8	94.3	93.8	94.6	94.2	93.9	97.7
	Segment 9	97.4	96.5	98.2	97.3	96.5	99.6
	Segment 10	93.8	94.5	92.9	93.7	94.8	97.2
	Segment 11	93.0	97.1	88.4	92.5	97.4	98.9
	Segment 12	92.1	90.5	93.8	92.1	90.4	96.5
	Average of all slices	93.2	92.9	93.7	93.2	92.9	97.3
Coronal	Segment 4	89.4	87.3	92.0	89.6	87.0	95.6
	Segment 5	94.3	92.3	96.4	94.3	92.2	98.1
	Segment 6	95.2	91.7	99.1	95.3	91.3	99.2
	Segment 7	91.2	91.1	91.1	91.1	91.3	96.2
	Segment 8	95.6	93.2	98.2	95.7	93.0	99.2
	Segment 9	94.3	95.4	92.9	94.1	95.7	98.5
	Segment 10	96.9	97.3	96.4	96.9	97.4	99.5
	Segment 11	89.0	88.5	89.3	88.9	88.7	94.9
	Segment 12	92.1	88.5	96.4	92.3	87.8	97.1
	Average of all slices	93.1	91.7	94.6	93.1	91.6	97.6
Sagittal	Segment 4	92.5	91.3	93.8	92.5	91.3	97.2
	Segment 5	89.4	87.9	91.1	89.5	87.8	96.5
	Segment 6	96.0	99.0	92.9	95.9	99.1	97.3
	Segment 7	94.3	93.8	94.6	94.2	93.9	98.2
	Segment 8	84.6	87.4	80.4	83.7	88.7	92.0
	Segment 9	91.2	88.3	94.6	91.4	87.8	97.5
	Segment 10	96.9	96.5	97.3	96.9	96.5	98.6
	Segment 11	95.6	94.7	96.4	95.6	94.8	97.9
	Segment 12	89.0	88.5	89.3	88.9	88.7	96.4
	Average of all slices	92.2	91.9	92.3	92.1	92.1	96.8

**Table 5:** AD vs. MCI.

Orientation	Slice Number	Accuracy	Precision	Sensitivity	Specificity	F1 Score	AUC
Axial	Segment 4	76.6	80.9	67.3	73.4	85.2	84.6
	Segment 5	83.4	78.9	89.4	83.8	77.9	93.2
	Segment 6	88.1	87.0	88.5	87.7	87.7	94.6
	Segment 7	88.9	88.5	88.5	88.5	89.3	94.0
	Segment 8	81.3	84.8	74.3	79.2	87.7	91.8
	Segment 9	83.8	87.9	77.0	82.1	90.2	93.2
	Segment 10	80.0	74.6	88.5	81.0	72.1	89.0
	Segment 11	81.3	76.3	88.5	82.0	74.6	92.3
	Segment 12	77.4	75.4	78.8	77.1	76.2	82.4
	Average of all slices	82.3	81.6	82.3	81.6	82.3	90.6
Coronal	Segment 4	90.2	90.9	88.5	89.7	91.8	94.6
	Segment 5	87.7	85.6	89.4	87.4	86.1	92.2
	Segment 6	88.5	95.7	79.6	87.0	96.7	95.6
	Segment 7	**92.3**	92.8	91.2	92.0	93.4	96.4
	Segment 8	69.4	65.0	78.8	71.2	60.7	79.3
	Segment 9	90.6	88.9	92.0	90.4	89.3	95.4
	Segment 10	87.2	88.1	85.0	86.5	89.3	92.3
	Segment 11	86.0	87.0	83.2	85.1	88.5	92.9
	Segment 12	74.9	76.5	69.0	72.6	80.3	82.5
	Average of all slices	85.2	85.6	84.1	84.6	86.2	91.3
Sagittal	Segment 4	86.0	81.7	91.2	86.2	81.1	90.7
	Segment 5	87.5	88.3	89.4	89.4	85.0	83.8
	Segment 6	88.1	87.0	88.5	87.7	87.7	93.1
	Segment 7	90.6	88.2	92.9	90.5	88.5	93.6
	Segment 8	85.1	81.5	89.4	85.2	81.1	93.7
	Segment 9	84.7	86.7	80.5	83.5	88.5	92.3
	Segment 10	89.4	82.8	98.2	89.9	81.1	97.5
	Segment 11	91.5	88.4	94.7	91.5	88.5	94.2
	Segment 12	90.6	91.0	89.4	90.2	91.8	95.9
	Average of all slices	84.2	76.4	80.5	78.3	87.6	90.1

**Table 6:** MCI vs. CN.

Orientation	Slice Number	Accuracy	Precision	Sensitivity	Specificity	F1 Score	AUC
Axial	Segment 4	86.5	89.5	83.6	86.4	89.6	93.8
	Segment 5	84.4	0.846	0.852	0.849	0.835	0.914
	Segment 6	85.2	92.2	77.9	84.4	93.0	91.9
	Segment 7	82.7	77.9	92.6	84.6	72.2	90.4
	Segment 8	85.2	84.3	87.7	85.9	82.6	90.8
	Segment 9	86.9	85.8	89.3	87.6	84.3	93.9
	Segment 10	82.7	79.1	90.2	84.3	74.8	90.2
	Segment 11	70.5	77.7	59.8	67.6	81.7	82.6
	Segment 12	75.5	76.2	76.2	76.2	74.8	82.7
	Average of all slices	82.2	83.0	82.5	82.4	81.8	89.7
Coronal	Segment 4	74.3	78.0	69.7	73.6	79.1	82.9
	Segment 5	81.4	84.2	78.7	81.4	84.3	91.5
	Segment 6	80.6	79.2	84.4	81.7	76.5	91.4
	Segment 7	77.2	87.8	64.8	74.5	90.4	86.7
	Segment 8	89.9	89.5	91.0	90.2	88.7	95.8
	Segment 9	83.5	82.2	86.9	84.5	80.0	92.2
	Segment 10	85.2	79.6	95.9	87.0	73.9	95.7
	Segment 11	76.8	71.6	91.0	80.1	61.7	87.5
	Segment 12	75.5	78.6	72.1	75.2	79.1	84.3
	Average of all slices	80.5	81.2	81.6	80.9	79.3	89.8
Sagittal	Segment 4	86.9	85.8	89.3	87.6	84.3	92.6
	Segment 5	81.4	81.5	82.8	82.1	80.0	91.2
	Segment 6	85.7	85.5	86.9	86.2	84.3	93.2
	Segment 7	81.4	85.5	77.0	81.0	86.1	89.5
	Segment 8	82.7	80.5	87.7	83.9	77.4	88.4
	Segment 9	84.8	80.7	92.6	86.3	76.5	92.4
	Segment 10	84.8	93.9	75.4	83.6	94.8	92.7
	Segment 11	86.5	88.8	84.4	86.6	88.7	92.5
	Segment 12	80.6	83.9	77.0	80.3	84.3	89.5
	Average of all slices	86.9	85.8	89.3	87.6	84.3	92.6

## Data Availability

Data are available at: https://adni.loni.usc.edu/ accessed on 11 July 2025.
